# Molecular Mechanisms for Ketone Body Metabolism, Signaling Functions, and Therapeutic Potential in Cancer

**DOI:** 10.3390/nu14224932

**Published:** 2022-11-21

**Authors:** Chi Yeon Hwang, Wonchae Choe, Kyung-Sik Yoon, Joohun Ha, Sung Soo Kim, Eui-Ju Yeo, Insug Kang

**Affiliations:** 1 Department of Biomedical Sciences, Graduate School, Kyung Hee University, Seoul 02447, Republic of Korea; 2 Biomedical Science Institute, Kyung Hee University, Seoul 02447, Republic of Korea; 3 Department of Biochemistry and Molecular Biology, School of Medicine, Kyung Hee University, Seoul 02447, Republic of Korea; 4 Department of Biochemistry, College of Medicine, Gachon University, Incheon 21999, Republic of Korea

**Keywords:** ketone bodies, β-hydroxybutyrate, ketogenic diet, inflammation, oxidative stress, post-translational modifications, cancer

## Abstract

The ketone bodies (KBs) β-hydroxybutyrate and acetoacetate are important alternative energy sources for glucose during nutrient deprivation. KBs synthesized by hepatic ketogenesis are catabolized to acetyl-CoA through ketolysis in extrahepatic tissues, followed by the tricarboxylic acid cycle and electron transport chain for ATP production. Ketogenesis and ketolysis are regulated by the key rate-limiting enzymes, 3-hydroxy-3-methylglutaryl-CoA synthase 2 and succinyl-CoA:3-oxoacid-CoA transferase, respectively. KBs participate in various cellular processes as signaling molecules. KBs bind to G protein-coupled receptors. The most abundant KB, β-hydroxybutyrate, regulates gene expression and other cellular functions by inducing post-translational modifications. KBs protect tissues by regulating inflammation and oxidative stress. Recently, interest in KBs has been increasing due to their potential for treatment of various diseases such as neurological and cardiovascular diseases and cancer. Cancer cells reprogram their metabolism to maintain rapid cell growth and proliferation. Dysregulation of KB metabolism also plays a role in tumorigenesis in various types of cancer. Targeting metabolic changes through dietary interventions, including fasting and ketogenic diets, has shown beneficial effects in cancer therapy. Here, we review current knowledge of the molecular mechanisms involved in the regulation of KB metabolism and cellular signaling functions, and the therapeutic potential of KBs and ketogenic diets in cancer.

## 1. Introduction

Ketone bodies (KBs) are a group of water-soluble molecules that contain the ketone groups, which consist of acetoacetate (AcAc), D-β-hydroxybutyrate (BHB), and acetone. Among KBs, BHB is the most abundant KB, accounting for about 70% of the circulating KB pool [1,2]. BHB and AcAc are important alternative energy sources for extrahepatic tissues during glucose deprivation and various physiological states, including fasting, starvation, neonatal period, and adherence to a very low-carbohydrate high-fat diet, which is termed a ketogenic diet (KD) [1,3,4]. 

KBs are produced by ketogenesis mostly in hepatocytes, but also to a minor extent in astrocytes, cardiomyocytes, renal epithelia, and enterocytes [5,6,7]. After the transport of fatty acyl-CoA to hepatic mitochondria via carnitine palmitoyl transferase 1/2 (CPT1/2), β-oxidation-derived acetyl-CoA usually condenses with oxaloacetate (OAA) to form citrate. However, increased fatty acid oxidation in the liver or low OAA levels due to gluconeogenesis can result in the formation of acetyl-CoA surpluses that can be used as substrates for ketogenesis [5,6]. KBs are secreted and transported to peripheral tissues via monocarboxylate transporters (MCT1, 2, and 4), also known as solute carrier 16A (SLC16A) family members, for ketolysis and ATP production in mitochondria [2,3,8,9,10]. Detailed pathways and regulatory mechanisms for ketogenesis and ketolysis are reviewed in Section 2. 

Under physiological conditions, plasma concentrations of KBs in adult humans range from 0.05–0.1 mM, rising to 1–2 mM after 2 days of fasting, and levels reaching 5–8 mM under prolonged fasting and starvation [1,2,11,12]. The condition in which there is an increase in blood KBs is called *ketonemia*, and a condition in which excretion in the urine is increased is called *ketonuria*. In general, ketonemia and ketonuria are collectively referred to as *ketosis*. However, in certain pathological conditions, such as diabetic ketoacidosis, KBs can exceed 20 mM. 

Although diabetic ketoacidosis is a pathological condition, mild ketonemia (nutritional ketosis) resulting from fasting, a KD, or other dietary interventions including calorie restriction (CR) has been demonstrated to be beneficial, improving metabolic profiles and extending healthy lifespan [13,14,15,16]. In addition to serving as energy fuel for extrahepatic tissues, KBs exert multifaceted beneficial effects by functioning as signaling molecules, protein post-translational modifiers, regulators of inflammation, oxidative stress, cellular senescence and aging [3,12,17,18]. KBs and KDs are of increasing interest due to their therapeutic potentials for various diseases including metabolic disorders, neurological and cardiovascular diseases, and cancer [3,19,20,21,22,23,24,25,26]. We will discuss the molecular mechanisms of the various cellular effects of KBs in Section 3.

Cancer cells undergo metabolic reprograming to maintain rapid cell proliferation and tumor growth [27,28,29]. A striking change in cancer cells is that glucose uptake increases even in the presence of oxygen and is subsequently fermented to lactate, which is called aerobic glycolysis and the Warburg effect [29,30]. Since cancer cells uptake and utilize more glucose than normal cells, diet-induced glucose reduction may help treat or prevent cancer [30,31,32]. KBs are increased during dietary restriction and mediate some anti-cancer effects of dietary interventions [2,33,34,35]. In addition, KB metabolism is dysregulated in various types of cancer and plays an important role in tumorigenesis [36,37,38,39,40,41]. Differentially expressed key enzymes of KB metabolism may serve as potential diagnostic and prognostic biomarkers and therapeutic targets. Furthermore, previous in vitro and in vivo investigations have demonstrated that KBs themselves have anti-tumor effects and that KBs can at least partially demonstrate the in vivo effects of dietary restriction [35,42,43,44]. Therefore, we are particularly interested in the effects of KBs and KD in cancer cell biology and cancer therapy. Here, in Section 4, we review the latest knowledge on the regulation of KB metabolism in cancer cells compared to normal cells. It also describes recent research reports on the molecular mechanisms for the beneficial effects of KBs and KD in anti-cancer therapies.

## 2. Pathways and Regulation of Ketogenesis and Ketolysis

### 2.1. Pathways of Ketogenesis and Ketolysis

**Figure 1:**
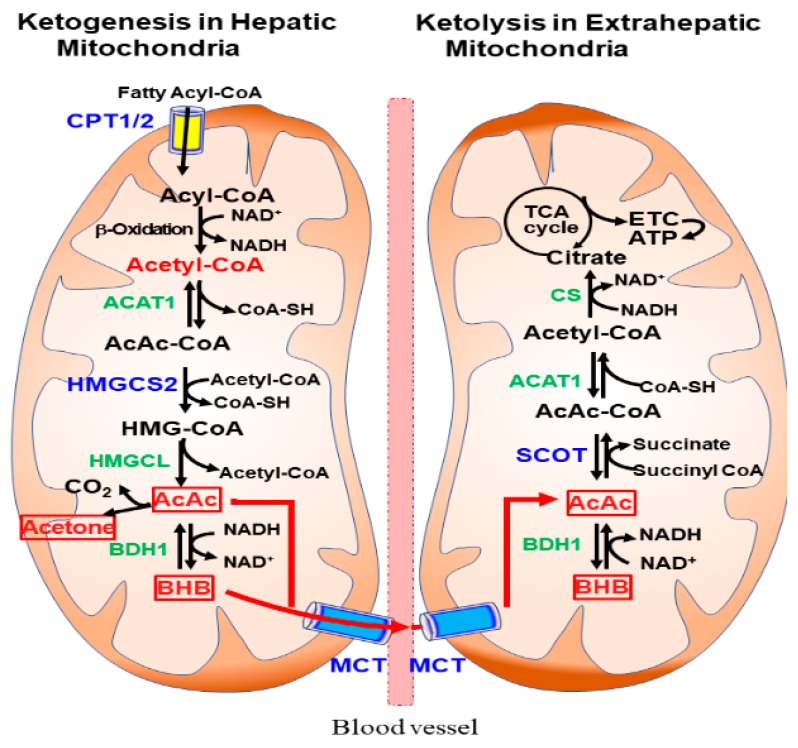
Ketogenesis and ketolysis pathways in mitochondria. Ketogenesis occurs predominantly in hepatic mitochondria using acetyl-CoA produced by β-oxidation of fatty acyl-CoA. After being taken up by extrahepatic tissues through monocarboxylic acid transporter (MCT), ketolysis occurs in the mitochondria, where β-hydroxybutyrate (BHB) and acetoactate (AcAc) are converted to Acetyl-CoA and ATP is produced via the tricarboxylic acid (TCA) cycle and electron transport chain (ETC).

The pathways of hepatic ketogenesis and extrahepatic ketolysis are shown in Figure 1. For ketogenesis, fatty acyl-CoA is transported to the hepatic mitochondria via CPT1/2 where β-oxidation occurs to produce acetyl-CoA. Two acetyl-CoA molecules first condense to form acetoacetyl (AcAc)-CoA in a reaction catalyzed by acetyl-CoA acetyltransferase 1 (ACAT1, also known as acetoacetyl-CoA thiolase) [4,5,6]. Ketogenesis proceeds further through a step-by-step reaction that is catalyzed by 3-hydroxy-3-methylglutaryl (HMG)-CoA synthase 2 (HMGCS2), HMG-CoA lyase (HMGCL), and β-hydroxybutyrate dehydrogenase 1 (BDH1). 

The ketone bodies AcAc and BHB are released into the circulation and taken up by extrahepatic tissues through MCTs. Ketolysis occurs in the extrahepatic mitochondria, where BHB is converted to AcAc by BDH1 and acetyl-CoA through a reaction catalyzed by succinyl-CoA:3-oxoacid-CoA transferase (SCOT) and ACAT1 [5]. After acetyl CoA is converted to citrate via citrate synthase (CS), ATP is produced through the tricarboxylic acid (TCA) cycle and the electron transport chain (ETC). In addition to ketolysis for ATP production, KBs can be utilized in de novo lipogenesis, sterol synthesis, and hexosamine biosynthesis in many tissues [1,4].

### 2.2. Regulation of Ketogenesis: The Role of HMGCS2 as a Rate Limiting Enzyme

Ketogenesis is regulated at several levels and the main regulatory points for ketogenesis include mobilization of free fatty acid, fatty acyl-CoA transport into mitochondria, and *HMGCS2* gene expression and activation of HMGCS2 protein, a key rate limiting enzyme in ketogenesis [5,6]. Mobilization of free fatty acid occurs due to lipolysis of triglycerides in adipocytes [5,45]. Fatty acyl-CoA transport to the mitochondria via CPT1 is increased when levels of malonyl-CoA, an allosteric inhibitor of CPT1A, are low [6]. 

**Figure 2:**
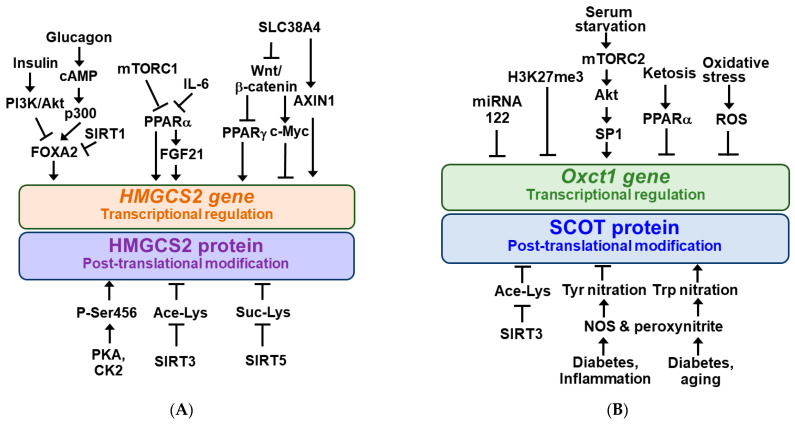
Regulatory mechanisms for HMGCS2 and SCOT. 3-Hydroxy-3-methylglutaryl-CoA synthase 2 (HMGCS2) (**A**) and succinyl-CoA:3-oxoacid-CoA transferase (*Oxct1*/SCOT) (**B**) gene expressions are regulated at the transcriptional levels and their protein activities are regulated by the post-translational modifications. Arrow (→) and truncated line (┬) indicate activation and inhibition, respectively.

*HMGCS2* gene expression is regulated at the transcriptional level and the activity of HMGCS2 protein is regulated at the post-translational level [4,6,46]. Regulators and signaling pathways for transcriptional and post-translational regulation of HMGCS2 are schematically shown in Figure 2A. *HMGCS2* gene transcription is regulated by insulin and glucagon ratios mediated by the transcription factor forkhead box A2 (FOXA2) [47]. Insulin signaling inactivates FOXA2 by phosphorylation and nuclear export via the phosphatidylinositol-3-kinase (PI3K)/Akt-dependent pathway [4,48]. In contrast, glucagon activates *HMGCS2* transcription by FOXA2 acetylation via the cAMP-p300-FOXA2 pathway, which is inhibited upon deacetylation of FOXA2 by the NAD^+^-dependent enzyme sirtuin 1 (SIRT1) [49]. 

*HMGCS2* gene expression is positively regulated by the peroxisome proliferator-activated receptor α (PPARα) [5]. Recent evidence suggests that PPARα stimulates *HMGCS2* gene expression in the liver through the expression of fibroblast growth factor 21 (FGF21), known as an endocrine regulator of ketotic states [5,6,50,51]. 

Meanwhile, *HMGCS2* transcription is negatively regulated by mechanistic target of rapamycin complex 1 (mTORC1) kinase [52,53] and tumor-derived interleukin-6 (IL-6) [54], presumably suppressing PPARα. mTORC1 is negatively regulated by AMP-activated protein kinase (AMPK), a metabolic master switch that regulates anabolic or catabolic metabolism depending on nutrient availability [5,55,56].

Recently, it has been reported that *HMGCS2* gene expression is positively regulated by PPARγ, which is inhibited by the Wnt/β-catenin signaling pathway in intestinal epithelial cancer cells [57]. SLC38A4 can increase HMGCS2 expression via inhibiting the Wnt/β-catenin/Myc axis and upregulating AXIN1 [58]. 

The HMGCS2 protein undergoes several different types of post-translational modifications, as shown in Figure 2A. HMGCS2 is a highly phosphorylated protein and serine 456 phosphorylation of HMGCS2 (P-Ser456) by protein kinase A (PKA) and/or casein kinase 2 (CK2) increases its enzymatic activity [59]. HMGCS2 activity is inhibited by acetylation at lysines 310, 447, and 473, and mitochondrial SIRT3 deacetylates HMGCS2 to increase its activity [60]. In addition, mitochondrial HMGCS2 activity in the liver is inhibited through succinylation of lysine residues and desuccinylation by mitochondrial SIRT5 increases its activity and ketogenesis [61].

### 2.3. Regulation of Ketolysis: The Role of SCOT as a Rate Limiting Enzyme

In the case of ketolysis, the main regulatory points include expression of the SCOT gene and activation of the SCOT protein, a key enzyme that catalyzes the rate-limiting step [45]. Two isoforms of SCOT gene have been identified: *Oxct1* and *Oxct2* [62]. It was shown that *Oxct1* mRNA levels in most cell lines were higher than those of the testis-specific isoform, *Oxct2*, suggesting that *Oxct1* may play a more dominant role than *Oxct2* in ketolysis [62,63]. The *Oxct1* gene product, OXCT1, is abundant in the brain, heart, and kidney, and can be detected in almost all extrahepatic tissues [62]. Like HMGCS2, *Oxct1* expression is regulated during transcriptional regulation and SCOT activity is regulated by post-translational modification. Their modulators and signaling pathways are schematically shown in Figure 2B. *Oxct1* gene expression in normal liver was inhibited by microRNA 122 during fetal-to-neonatal transition and trimethylation of histone H3 at lysine 27 (H3K27me3) [4,64,65]. Therefore, in normal hepatocytes, the ketolytic enzyme SCOT is not expressed, which interferes with the use of KB. In contrast, serum starvation in hepatocellular carcinoma (HCC) cells increases OXCT1 expression through activation of the mTORC2-Akt-SP1 pathway [38]. Persistent ketosis prevented KB utilization by suppressing *Oxct1* mRNA and SCOT protein expression in the heart and muscle of animals, presumably via PPARα [3,66]. It was also reported that *Oxct1* mRNA and SCOT protein expression was decreased in the failing heart myocardium and cardiomyocytes by oxidative stress [7]. 

The SCOT protein was found to be hyperacetylated in the brain of SIRT3 knockout mice, which correlated with decreased ketolytic capacity [4,67]. SCOT was shown to be susceptible to tyrosine (Tyr) nitration in the hearts of streptozotocin-treated (diabetic) or endotoxin-exposed (inflammatory) rats, and the catalytic activity of Tyr-nitrated SCOT is reduced [68,69]. SCOT was also nitrated at tryptophan (Trp) residues in aged and diabetic animals and increased the specific activity of Trp-nitrated SCOT [70,71]. Nitric oxide produced by nitric oxide synthase (NOS) and subsequent intramitochondrial formation of peroxynitrite (ONOO^−^) may be responsible for nitration modifications of SCOT proteins [68,71].

## 3. Molecular Mechanisms of Beneficial Effects of Ketone Bodies

### 3.1. Ketone Bodies as Energy Fuels for Extrahepatic Tissues 

The main function of KBs is to provide extrahepatic tissues with the energy they need more efficiently during nutrient deprivation [72,73]. In particular, BHB has been shown to produce more ATP per oxygen molecule consumed than pyruvate, and BHB increases the Gibbs free energy change for ATP hydrolysis compared to glucose [74]. Importantly, some of the therapeutic applications of KBs are well related to their role in energy metabolism [22,74]. For example, KDs and KBs are used in the treatment of glucose transporter 1 (GLUT1) and pyruvate dehydrogenase deficiency syndrome by providing an alternative energy source that bypasses defects in glucose metabolism [6,75]. During aging and neurodegenerative diseases such as Alzheimer’s disease and Parkinson’s disease, KBs can become an important energy source for the brain to ameliorate its energy crisis [76,77]. 

### 3.2. Ketone Bodies as Anti-Inflammatory Signaling Molecules

**Figure 3:**
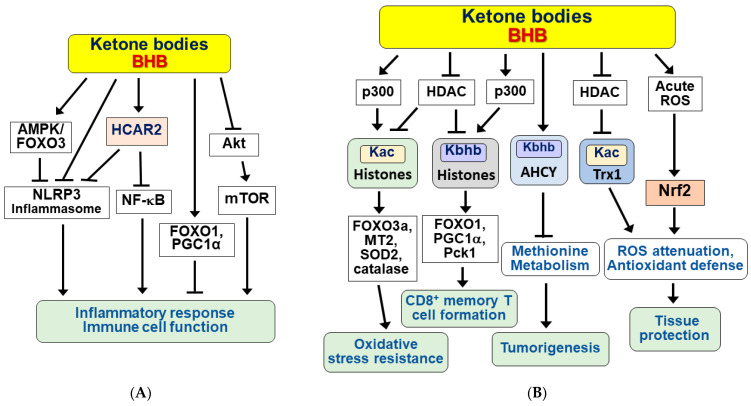
Signaling functions of ketone bodies for anti-inflammation, epigenetic and post-translational modifications, and antioxidative stress. Ketone bodies (KBs) including BHB exert a variety of beneficial effects. (**A**) KBs exhibit anti-inflammatory responses and modulate immune cell functions in a receptor (HCAR2)-dependent and independent manner. (**B**) KBs regulate various cellular processes by inducing epigenetic and post-translational modifications of histone and non-histone proteins and by reducing oxidative stress. Arrow (→) and truncated line (┬) indicate activation and inhibition, respectively.

KBs exert many beneficial effects, such as protection against renal and hepatic ischemia/reperfusion injury, chronic inflammation-induced cardiovascular diseases, and diabetic retinal damage, by functioning as anti-inflammatory signaling molecules [4,78,79,80,81]. BHB inhibits inflammatory responses and immune cell function either by binding to and activating hydroxycarboxylic acid receptor 2 (HCAR2) or directly modulating some intracellular signaling pathways [82,83]. The molecular mechanism is diagramed in Figure 3A.

HCAR2 is a Gi/o type of cell surface G-protein coupled receptors (GPCRs) and is abundantly expressed in various cells, including macrophages, monocytes, dendritic cells, adipocytes, and epithelial cells [84,85]. The BHB/HCAR2 signaling pathway suppresses the inflammatory response by inhibiting nuclear factor-kappa B (NF-κB) [86,87,88]. Interestingly, BHB blocks activation of the NOD-like receptor pyrin domain-containing protein 3 (NLRP3) inflammasome in a HCAR2-dependent or independent manner, depending on cell type and inflammatory stimuli [81,89,90]. BHB was also shown to inhibit endoplasmic reticulum (ER) stress-induced NLRP3 inflammasome in aged rats via the AMPK-forkhead box transcription factor O3 (FOXO3) pathway [91]. In addition, BHB was shown to exert anti-inflammatory effects through the selective interaction of FOXO1 with PPARγ coactivator 1α (PGC-1α) in the kidneys of aged rats [92]. BHB also exerted anti-inflammatory effects through inhibition of the Akt-mTOR signaling pathway in MIN6 pancreatic β cells [93].

Although BHB was associated with anti-inflammatory effects in most studies [87,88,89,92,94], KBs or ketone supplementation was also associated with pro-inflammatory effects and increased pro-inflammatory factors and cytokines [95,96,97,98]. The anti- and pro-inflammatory effects of KBs may vary with cell type, concentration, and duration of exposure. Nevertheless, anti-inflammatory responses may be one of the mechanisms for the beneficial effects of KBs. 

### 3.3. Ketone Bodies as Epigenetic and Post-Translational Modifiers of Histones and Nonhistone Proteins

KBs, including BHB, exert beneficial effects in part by regulating gene expression through epigenetic and post-translational modifications of histones and nonhistone proteins [99,100]. Some of the BHB-induced modifications, including acetylation and β-hydroxybutyration, downstream signaling pathways, and physiological effects are schematically shown in Figure 3B. BHB inhibits histone deacetylase (HDAC) 1, 3, and 4 (classes I and IIa) in vitro and in vivo, at multiple sites including H3 lysines 9 and 14 (H3K9ac, H3K14ac) [101]. BHB-induced histone hyperacetylation is associated with global changes in transcription, including induction of oxidative stress resistance genes such as FOXO3a, metallothionein 2 (MT2), superoxide dismutase 2 (SOD2), and catalase. However, in other studies, BHB did not significantly affect HDAC activity and histone acetylation, but rather lysine β-hydroxybutyrylation (Kbhb) of histones [102,103]. HDAC1 and HDAC2 can remove Kbhb, whereas the well-known acetyltransferase p300 catalyzes the enzymatic addition of BHB to lysine [104]. It has recently been shown that BHB induces the Kbhb of histone H3K9, leading to upregulation of FOXO1 and PGC1α as well as phosphoenolpyruvate carboxykinase (Pck1), a target gene involved in glycogen metabolism and formation and maintenance of CD8^+^ memory T cells [105]. 

Interestingly, nonhistone proteins also undergo β-hydroxybutyrylation [106,107] and acetylation [108]. S-adenosylhomocysteine hydrolase (AHCY), a rate-limiting enzyme of the methionine cycle, is lysine β-hydroxybutyrylated to inhibit enzymatic activity under ketogenic conditions [106]. Since cancer cells depend on methionine metabolism [109], BHB may exert anti-cancer effects in tumors via Kbhb of AHCY. It was also reported that p53 Kbhb levels were significantly increased in BHB-treated cultured cells and thymus tissues of fasted mice [107]. The p53 Kbhb modification lowered the level of p53 acetylation and decreased p53 transcriptional activity, suggesting that BHB may interfere with the function of wild type p53. Similarly, BHB increases thioredoxin 1 (Trx1) acetylation and protein stabilization through inhibition of HDAC1 expression, resulting in Trx1 upregulation and antioxidant defense [108].

### 3.4. The Protective Effect of Ketone Bodies against Oxidative Stress-Induced Damage

An important regulatory function of KBs is its effect on reactive oxygen species (ROS) metabolism and maintenance of redox homeostasis. The antioxidant effects of KBs have been widely studied both in vitro and in vivo [3,110,111,112,113]. KBs reduce oxidative stress-induced cell damage and apoptosis by attenuating ROS and enhancing antioxidant defense in neurons and cardiomyocytes [7,108,110,111,114,115]. 

BHB can act as a direct antioxidant against hydroxy radicals [110]. BHB can oxidize coenzyme Q couple, decreasing the amount of semiquinone in coenzyme Q [20,74]. BHB also protects against oxidative stress by promoting the transcriptional activity of nuclear factor erythroid 2-related factor (Nrf2) and target genes for antioxidant defense [116]. BHB generated with CR or BHB supplementation inhibited ischemic retinal degeneration through upregulation of Nrf2 [117]. However, conflicting reports suggest that KBs induce oxidative stress in a variety of cells, including cardiomyocytes, smooth muscle cells, endothelial cells, and hepatocytes [95,118,119,120,121]. Similarly, KD administration was shown to induce acute oxidative stress in mitochondria and modulate redox signaling by Nrf2 [122]. Nevertheless, KB-induced oxidative stress may be beneficial as it can initiate adaptive responses by activation of master regulators of cytoprotection, including Nrf2, SIRT1 and 3, and AMPK [116,123]. 

## 4. Ketone Bodies in Cancer Cell Biology and Cancer Therapy

### 4.1. Dysregulation of Ketone Body Metabolism in Cancer Cells 

Metabolic reprograming of cancer cells is associated with their rapid growth and proliferation [27,28,29]. The Warburg effect arises from the dominant role of glycolysis. Cancer cells favor increased glycolysis over oxidative phosphorylation because glycolysis generates both biosynthetic precursors required to support cancer cell proliferation and glutathione to combat oxidative stress [29,30]. 

**Figure 4:**
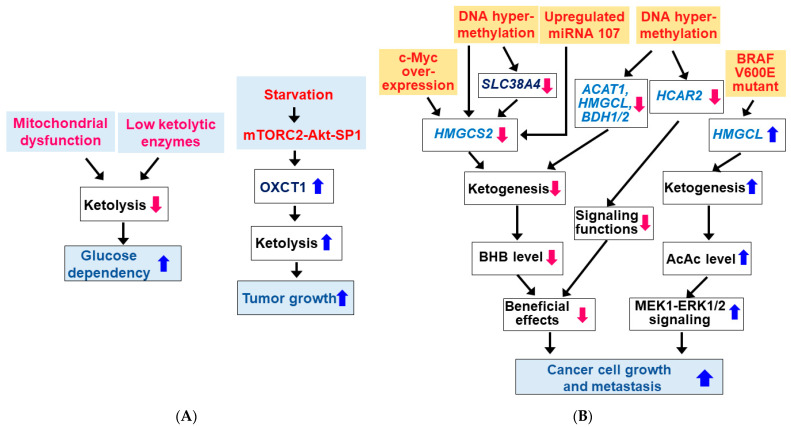
Dysregulation of ketone body metabolism in cancer cells. (**A**) Changes in ketolysis in cancer cells. Many tumor cells are inefficient in KB utilization due to mitochondrial dysfunction and/or low ketolytic enzymes. (**B**) Changes in ketogenesis in cancer cells. Expressions of ketogenic enzymes and HCAR2 are altered in some cancer cell types. Blue (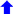) and red (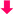) arrows indicate increases and decreases, respectively.

During glucose starvation, normal cells use KBs as metabolic fuel. It has been shown that many tumor cells are inefficient at utilizing KBs due to dysfunction of mitochondria and/or deficient ketolytic enzymes (Figure 4A) [44,124,125,126]. It is reported that tumor cells can use KBs as precursors for lipid synthesis rather than as energy substrates [5,127]. However, some cancer cells have normal mitochondria and can metabolize KBs by expressing ketolytic enzymes [124,128,129]. As previously described, the ketolytic enzyme OXCT1 is not expressed in normal hepatocytes but is upregulated by serum starvation via activation of the mTORC2-Akt-SP1 pathway in HCC cells (Figure 4A) [38]. The SCOT activity may play a role in tumor growth and metastasis [38,62,130]. KB utilization is an indication of metabolic flexibility of cancer cells, and KB oxidation generally provides a growth advantage during glucose deprivation [5]. 

The production of KBs by cancer cells has been shown to inhibit or promote the growth and proliferation of cancer cells. Therefore, questions about the importance of ketogenesis in cancer cells have been raised. Here, we review how key enzymes/proteins involved in KB metabolism and signaling pathways are altered in cancer cells. Interestingly, the expression and activity of HMGCS2 was shown to decrease in cancer cells, resulting in low BHB production (Figure 4B). Expression of HMGCS2 was reduced during cancerous transformation via c-Myc overexpression in colonic epithelium [131]. HMGCS2 is a direct target for c-Myc and c-Myc represses *HMGCS2* transcription and its protein expression in c-Myc-dependent colon and rectal tumors. Consistently, HMGCS2 protein expression was downregulated in colorectal cancer patients [132]. Similarly, HMGCS2 was downregulated in HCC tissues and lower HMGCS2 expression was correlated with higher morbidity grade and stage [58,133,134]. HMGCS2 overexpression or BHB supplementation inhibited liver cancer cell growth and migration. In addition, reduced transcription of the *HMGCS2* gene by DNA hypermethylation has been shown to reduce ketogenesis and facilitate tumor cell motility in clear cell renal cell carcinoma (ccRCC) [135]. Similar to HMGCS2, SLC38A4 expression was downregulated in fetal liver and HCC due to DNA hypermethylation and low expression of SLC38A4 was associated with poor prognosis in HCC patients [58]. Because SLC38A4 increased HMGCS2 expression by repressing the Wnt/β-catenin/MYC axis and upregulating AXIN1, DNA hypermethylation and thus downregulation of SLC38A4 may also be responsible for the downregulation of HMGCS2 in HCC. HMGCS2 protein expression was also downregulated by miRNA107 targeting the 3′-UTR of *HMGCS2* mRNA in HCC [133]. HMGCS2 protein expression was also reduced in prostate cancer tissues and low mRNA expression was associated with high pathological grade as well as the presence of distant metastases [41]. HMGCS2 knockdown promoted cell proliferation, invasion, and migration of prostate cancer cells. 

Similarly, it was shown that other ketogenic enzymes such as ACAT1, HMGCL and/or BDH1/2 levels were significantly downregulated in ccRCC cells and patients [36], acute myeloid leukemia (AML) patients and AML cell lines [37], and nasopharyngeal carcinoma (NPC) cells [40,136]. Similar to HMGCS2, DNA hypermethylation may also play a role in downregulation of these ketogenic enzymes [40]. Low expression of these genes was associated with a worse prognosis and outcome in cancer patients. Since overexpression of each of these genes inhibited growth/proliferation and migration/metastasis of these cancer cells, genes for ketogenic enzymes such as *HMGCS2*, *ACAT1*, *HMGCL*, and *BDH* are suggested to be potential tumor suppressors.

Expression of HCAR2 has shown to be suppressed in colon and mammary tumors due to epigenetic mechanisms involving DNA hypermethylation [137,138]. Targeted deletion of the DNA methyltransferase 1 (DNMT1) induced HACR2 expression in colon cancer cell lines, suggesting that DNMT1 is responsible for the silencing of HCAR2 in colon cancer [138]. Moreover, re-expression of HCAR2 by cDNA transfection in colon cancer lines induced apoptosis only in the presence of the ligand. This suggests that circulating BHB produced during fasting or a KD exerts a cancer-preventive effect through HCAR2 activation [139]. HCAR2 may also function as a tumor suppressor.

In contrast, it was reported that HMGCL expression was upregulated in oncogenic mutant BRAF V600E-expressing cancer cells and the increased AcAc promoted activation of mitogen-activated protein kinase kinase 1 (MEK1)-extracellular signal-regulated kinase 1/2 (ERK1/2) signaling that drives tumor cell proliferation and growth [39]. A recent study also showed that HMGCL was upregulated in human pancreatic ductal adenocarcinoma (PDAC) and that depletion of HMGCL impeded migration and invasiveness and reduced tumor cell growth in vivo [140]. In this case, BHB was metabolized and stimulated metastasis, suggesting that HMGCL and BHB contribute pancreatic tumor aggressiveness. Other studies have also shown that cancer cell ketogenesis and subsequent ketolysis may have protumor/oncogenic effects. Increased expression of both ketogenic and ketolytic enzymes including ACAT1 and OXCT1 was reported in human tissues of aggressive and castrate-resistant prostate cancer [141,142,143]. Similarly, the protumor effect of enzymes of ketone body metabolism was also seen in breast cancer cells. In human breast cancer tumor samples, ketogenic enzymes were preferentially expressed in stroma and KBs produced by catabolic fibroblasts promoted tumor growth of adjacent breast cancer cells [144]. 

Taken together, dysregulated ketone body metabolism and its significance in cancer cells may vary depending on the cell type, grade and stage of the tumor, and the genetic mutation. 

### 4.2. Beneficial Effects of Ketone Bodies and Ketogenic Diet in Cancer Therapy

Since cancer cells prefer glycolysis and use more glucose than normal cells, diets targeted to the Warburg effect can starve cancer cells by reducing blood glucose levels [30,145,146]. Consistent with this concept, dietary interventions such as CR, fasting, and fasting-mimicking diet, and a KD have shown beneficial effects in cancer treatment [31,32,147,148,149,150,151,152,153]. Diet restriction with CR and fasting has been shown to selectively sensitize cancer cells to standard cancer therapies such as chemotherapy and radiation, while protecting normal cells from the side effects of cytotoxic treatments [147,154,155]. CR and fasting may function by promoting a wide range of changes in metabolic pathways and cellular functions, including an increase in stress responses and a decrease in insulin-like growth factor-1 (IGF-1) and other growth factors that stimulate cancer cell growth and proliferation [147]. Additionally, KBs produced during fasting may help slow tumor growth and promote cell differentiation [147]. Evidence for the anti-cancer effects of KBs came from data showing that ketone supplementation alone was effective in some cancer models [25,35,43]. Due to the difficulty in adapting to KD and fasting, the use of ketone supplements such as ketone salts, 1,3-butanediol, and ketone esters is currently being evaluated as a way to reach ketosis without dietary restriction [30,35,156,157]. KBs have been shown to exert growth inhibitory and cytotoxic effect in various cancer cell lines including lymphoma, cervical cancer, melanoma, glioblastoma, neuroblastoma, and pancreatic cancer cells [35,42,43,44].

KDs lower blood glucose and provide KBs to tissues, thereby reducing cancer cell proliferation, and enhancing survival. KD alone or in combination with CR has been successful in the treatment of malignant gliomas in animal models [126,152,158] and in patients with brain tumors [159,160,161,162,163]. Tumor-suppressive effect and anti-cancer progression by the KD has also been shown in many other cancer models including colon, lung, neuroblastoma, breast, pancreas, prostate, and stomach cancer [148,164,165,166,167,168,169,170,171]. 

Although a KD provides anti-tumor benefits on its own for certain types of cancer, it is much more effective when combined with chemotherapy and radiation [26,34,148,154,172,173,174]. For an example, the combination of a KD and chemotherapy synergistically inhibited tumor growth, resulting in a 3-fold increase in the survival benefit of chemotherapy alone in an animal model of pancreatic cancer [175]. A KD has been shown to enhance the efficacy of targeted therapy, such as PI3K inhibitors and to overcome drug resistance in mouse models of various cancer types, including pancreatic, bladder, endometrial, breast, and AML [176]. In addition, a KD can enhance the efficacy of anti- programmed cell death 1 (PD-1) immunotherapy in animal models of RET melanoma, renal cell carcinoma, and lung cancer [26] and anti- cytotoxic T lymphocyte-associated protein-4 (CTLA-4) immunotherapy in breast and colon cancer cells and improved overall survival in mouse tumor models [177].

In contrast, a KD showed the protumor effect in other studies including kidney cancer in a rat model of tuberous sclerosis [178]. It was also reported that KD increased BHB levels but had no effect on tumor growth and survival in rat glioma models, and transplanted brain tumors oxidized BHB to levels similar to those in the contralateral brain [128]. It has also been shown that breast cancer cells expressing MCT2 can uptake BHB and increase tumor cell malignancy in vitro and in vivo [179]. Consistent with the protumor effect of BHB, AcAc produced by upregulated HMGCL or KD promoted tumor cell growth and proliferation in BRAF V600E-expressing melanoma [39,180]. 

However, a recent study showed that in mice bearing BRAF V600E mutant and NRAS Q61R mutant xenografts, KBs had no direct effect on melanoma cell growth, whereas a KD slowed tumor [181]. This suggests that the KD induces anti-tumor effects toward melanoma regardless of genetic background and metabolic plasticity. Therefore, it is suggested that numerous factors control the ability of cells to utilize KBs in vitro and in vivo, and their sensitivity to KD’s or KBs’ action may vary with cancer type, grade and stage, and genetic mutations [5,148,171]. 

Collectively, extensive studies support the beneficial effects of KDs in reducing tumor growth, preventing cancer, enhancing the efficacy of cancer therapies, reducing the side effects of cytotoxic treatments, and increasing survival [25,26,32,145,148]. Moreover, a KD in cancer patients appears to be safe and feasible as an adjuvant therapy with beneficial effects on body composition, physical function, and quality of life [100,155,182,183]. 

### 4.3. Potential Mechanisms for Ketone Bodies and Ketogenic Diets in Cancer Therapy

KBs and a KD appear to show therapeutic potential in cancer treatment [145,184], but preclinical studies have shown that all tumors do not respond in the same way [100,182]. Differential response to KBs and a KD may be justified by the ability of tumor cells to use KBs as metabolic fuel via ketolysis [63,185]. Cells with low levels of the ketolytic enzymes BDH1 and OXCT1 were more responsive to BHB and KDs in cancer cells in vitro and in vivo [63]. 

**Figure 5:**
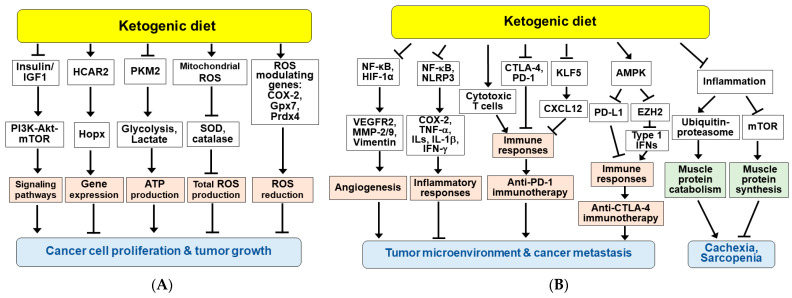
Potential mechanisms for the beneficial effects of ketogenic diet and/or ketone bodies in cancer therapy. KDs exert anti-tumor effects through a variety of mechanisms, including elevated KB levels and decreased blood glucose. (**A**) KDs and/or KBs inhibit cancer cell proliferation and tumor growth. (**B**) KDs and/or KBs improve the tumor microenvironment and suppress cancer metastasis, cachexia, and sarcopenia. Arrow (→) and truncated line (┬) indicate activation and inhibition, respectively.

Potential mechanisms for KBs and KDs in cancer therapy are schematically shown in Figure 5. KD can affect tumor cell growth by lowering insulin and IGF-1 levels, thereby reducing receptor tyrosine kinase-dependent signaling pathways such as PI3K-Akt-mTOR for tumor cell proliferation and tumor growth (Figure 5A) [154,158,171,176]. A recent study showed that a KD, BHB, or ketone supplementation exhibited a strong tumor-suppressive effect in an animal model of colorectal cancer [25]. BHB supplementation reduced proliferation of colonic crypt cells and inhibited intestinal tumor growth through HCAR2 activation and induction of transcriptional regulator Hopx, thereby altering gene expression and inhibiting cancer cell proliferation. Similarly, the anti-tumor properties of the KD correlated with downregulation of expression levels of pyruvate kinase M2 (PKM2), a key rate-limiting enzyme of glycolysis, in CT26^+^ colon cancer mouse model [186,187]. BHB also inhibited glycolysis by reducing the expression of PKM2 [188].

KBs and/or KDs have been shown to increase ROS levels in cancer cells. BHB and KD reduced cell proliferation and induced apoptosis through ROS production in glioma-stem-like cells [188] and CT26^+^ colon cancer model [186]. Therefore, KBs and KDs can augment conventional oxidative therapies such as radiation and chemotherapy [174,188]. However, it was also reported that the KD reduced ROS levels in gliomas, accompanied by slowed tumor growth and changes in ROS modulating genes, including cyclooxygenase-2 (COX-2), glutathione peroxidase 7 (Gpx7) and peroxiredoxin 4 (Prdx4) [158]. CR and KD can enhance DNA repair in normal tissues by increasing SIRT1 activity and decreasing PI3K-Akt signaling, but not in tumors [154]. BHB may modulate antioxidant defense programs and maintain redox homeostasis, which may contribute to the beneficial effect of dietary restriction on enhanced stress resistance [17,33].

Administration of KDs in mouse models has been shown to enhance the anti-angiogenic efficacy of cancer therapies [189,190]. KDs ameliorated tumor hypoxia environment and reduced tumor microvasculature in mouse glioma models, which was associated with inhibition of NF-κB and hypoxia inducible factor-1 (HIF-1) and their target genes such as vascular endothelial growth factor receptor 2 (VEGFR2), matrix metalloproteinase-2 (MMP-2) and vimentin (Figure 5B) [190].

Inflammation is associated with tumor progression and resistance to cancer treatment [191]. Cancer cells and surrounding stromal and immune cells can form the tumor microenvironment. Combination cancer therapy with KD showed decreased expression of COX-2 and pro-inflammatory cytokines, such as tumor necrosis factor-α (TNF-α), interferon-γ (IFN-γ), and ILs including IL-1β) via inhibition of NF-κB [169,172]. The anti-inflammatory effects of KBs and a KD may improve response to cancer treatment and prevention [146]. BHB has also been shown to inhibit glioma cell migration by inhibiting the NLRP3 inflammasome, suggesting that BHB may reduce the inflammatory microenvironment, providing an ancillary therapeutic benefit to the intervention of gliomas [192].

KDs and KBs have been shown to affect the immune system and reverse tumor-mediated immune suppression in malignant tumors [26,193]. KDs increased tumor-reactive immune responses, including tumor infiltration of cytotoxic CD8^+^ T cells in malignant glioma mouse model and reduced the expression of T cell co-inhibitory receptors CTLA-4 and PD-1 and their ligands CD86 and PD-L1 [193]. A recent study reported that a KD and BHB attenuated the KLF5-dependent production of CXCL12, thereby reducing colorectal cancer metastasis and enhancing the anti-tumor efficacy of anti-PD1 immunotherapy [132]. These suggest that a KD and BHB improve the immunosuppressive tumor microenvironment and enhance the anti-tumor effects of immunotherapy. In addition, a recent study showed that AMPK induced by a KD enhanced the efficacy of anti-CTLA-4 immunotherapy by decreasing PD-L1 and increasing the expression of type-1 IFN and antigen presenting genes in breast and colon cancer cells [177]. AMPK is activated by energy-deficient conditions such as fasting and KDs, and phosphorylates PD-L1 and EZH2, resulting in PD-L1 degradation and enhancement of IFNs expression, respectively.

Finally, in addition to anti-tumor effects, KBs have shown to suppress protein catabolism during starvation and LPS-stimulated inflammatory condition [194,195]. The anti-catabolic effects of BHB were associated with anti-inflammatory effects and regulation of protein turnover through ubiquitin proteasome-mediated muscle protein breakdown and mTOR-mediated muscle protein synthesis (Figure 5B) [196]. Consistently, KBs and KDs have been shown to prevent cachexia in patients undergoing chemotherapy and a ketone supplementation attenuated wasting in models of atrophy [148,196,197,198]. These suggest that KD may be an alternative dietary approach for cancer patients at risk of cachexia and sarcopenia.

## 5. Conclusions

The expression and activity of key rate-limiting enzymes for ketone body metabolism are regulated by transcriptional regulation and post-translational modifications. In addition to their role as metabolic fuels in extrahepatic tissues, KBs can exert many other beneficial effects such as neuroprotection, cardioprotection, anti-aging, and anti-cancer. KBs serve as signaling molecules regulating inflammation and immune function through receptor-dependent and independent pathways. KBs also exert beneficial effects by inhibiting ROS production and regulating gene expression and other cellular functions through post-translational modifications of histone and nonhistone proteins. Through these effects, KBs have a high therapeutic potential for various diseases including cancer.

Cancer cells reprogram glucose metabolism, such as the Warburg effect, for rapid cell proliferation and tumor growth. KB metabolism is also dysregulated in many types of cancer. KDs may exert anti-tumor effects through a variety of mechanisms, including elevated KBs and decreased blood glucose. Data from preclinical and clinical trials have shown strong support for the use of KDs in preventive and adjuvant cancer therapy. However, the molecular mechanism involved in the regulation of cancer biology by KBs is not fully understood. Further studies are needed to clarify the roles and mechanisms of KBs and KDs in various cancer types and cancer treatment regimens.

## Data Availability

Not applicable.

## Abbreviations

AcAc: acetoacetate; ACAT1, acetyl-CoA acetyltransferase 1; AHCY, S-adenosylhomocysteine hydrolase, AML, acute myeloid leukemia; AMPK, AMP-activated protein kinase; BDH1, β-hydroxybutyrate dehydrogenase 1; BHB, β-hydroxybutyrate; ccRCC, clear cell renal cell carcinoma; CK2, casein kinase 2; COX-2, cyclooxygenase 2; CPT1, carnitine palmitoyl transferase 1; CR, calorie restriction; CS, citrate synthase; CTLA-4, cytotoxic T lymphocyte-associated protein-4; CXCL12, C-X-C motif chemokine ligand 12; DNMT1, DNA methyltransferase 1; ER, endoplasmic reticulum; ERK1/2, extracellular signal-regulated kinase 1/2; ETC, electron transport chain; EZH2, enhancer of zeste homolog 2; FGF21, fibroblast growth factor 21; FOXA2, forkhead box A2; FOXO3, forkhead box O3; GLUT1, glucose transporter 1; GPCR, G-protein coupled receptor; Gpx7, glutathione peroxidase 7; HCAR2, hydroxycarboxylic acid receptor 2; HCC, hepatocellular carcinoma; HDAC, histone deacetylase; HIF-1, hypoxia inducible factor-1; HMG-CoA, 3-hydroxy-3-methylglutaryl-CoA; HMGCL, 3-hydroxy-3-methylglutaryl-CoA lyase; HMGCS2, 3-hydroxy-3-methylglutaryl-CoA synthase 2; IGF-1, insulin-like growth factor 1; IFN-γ, interferon-γ; IL-6, interleukin-6; KB, ketone body; KD, ketogenic diet; KLF5, Krüppel-like factor 5; MCT, monocarboxylate transporter; MEK1, mitogen-activated protein kinase kinase 1; MMP-2; matrix metalloproteinase-2; MT2, metallothionein 2; mTOR, mechanistic target of rapamycin; mTORC1, mTOR complex 1; NF-κB, nuclear factor kappa B; NLRP3, NOD-like receptor pyrin domain-containing protein 3; NOS, nitric oxide synthase; NPC, nasopharyngeal carcinoma; Nrf2, nuclear factor erythroid 2-related factor 2; OAA, oxaloacetate; Pck1, phosphoenolpyruvate carboxykinase 1; Oxct1, 3-oxoacid-CoA transferase 1; PD-1, programmed cell death-1; PDAC, pancreatic ductal adenocarcinoma; PGC-1α, peroxisome proliferator activated receptor γ coactivator 1α; PI3K, phosphatidylinositol-3-kinase; PKA, protein kinase A; PKM2, pyruvate kinase M2; PPARα, peroxisome proliferator activated receptor α; Prdx4, peroxiredoxin 4; ROS, reactive oxygen species; SCOT, succinyl-CoA:3-oxoacid-CoA transferase; SIRT, sirtuin; SLC16A, solute carrier 16A; SOD2, superoxide dismutase 2; TCA, tricarboxylic acid; TNF-α, tumor necrosis factor-α; Trx1, thioredoxin 1; VEGFR2, vascular endothelial growth factor receptor 2.
